# Effects of long-term care insurance on health: a study of the middle-aged and older adult in pilot cities in China

**DOI:** 10.3389/fpubh.2025.1498105

**Published:** 2025-05-19

**Authors:** Qian Chen, Sang Ma, Xinyue Lyu

**Affiliations:** ^1^School of Government, Yunnan University, Kunming, China; ^2^School of Humanities and Social Sciences, Guangxi Medical University, Nanning, China

**Keywords:** long-term care insurance (LTCI), long-term care (LTC), policy evaluation, difference-in-difference model, health performance

## Abstract

**Background:**

China established a trial long-term care insurance program in 15 cities in 2016 to address the country’s aging population; however, the policy’s impacts must be confirmed.

**Methods:**

Panel data from the China Health and Retirement Longitudinal Study (CHARLS) were used, covering four periods: 2011, 2013, 2015, and 2018. A difference-in-difference model was applied to analyze the impact of the long-term care insurance policy on health status, comparing residents in pilot cities with those in non-pilot cities.

**Results:**

The implementation of long-term care insurance in pilot cities led to a significant increase in self-rated health of 0.093 levels among surveyed residents compared to those in non-pilot cities. Significant variables included educational attainment, household registration, marital status, and the regional number of hospitals. Additionally, the health condition of citizens in central and western pilot cities improved considerably compared to those in eastern pilot cities.

**Conclusion:**

The long-term care insurance policy has a significant positive effect on the health status of residents, demonstrating its potential as a valuable policy tool to address health challenges associated with an aging population.

## Introduction

Unlike the risks of old age—illness and accidents—almost everyone has varying levels of incapacity in old age. The higher the life expectancy, the longer the period of disability survival. China is facing a significant challenge because of its rapidly aging population due to an increasing average life expectancy and declining fertility rates. The demand for long-term care for frail and disabled older adult individuals is increasing due to the rapid rise in the aging population (1, 2). Simultaneously, as women seek equal status and employment rates increase, fewer women are willing to stay at home to care for the older adult (3). In addition, the fertility policy implemented in China in the past has led to smaller family sizes and the loss of the old model of care-sharing between multiple children; informal care from the family is no longer sufficient to meet real needs (4). Consequently, families and individuals need a protective mechanism to share long-term care responsibilities and the high costs associated with incapacity (5).

Long-term care insurance (LTCI) is one way to meet the demand for disability care in an aging society and alleviate the heavy financial and mental burden of caregiving for disabled families (6). Currently, some developed countries that entered an aging society earlier have successfully promoted this system (7, 8), effectively solving the financial risks associated with long-term care of the older adult. The implementation of long-term care insurance (LTCI) holds profound significance, serving not only as a crucial strategy to address the challenges posed by an aging population and the increasing number of incapacitated older adult individuals but also as a pivotal component in the enhancement of China’s social security system. By covering the costs associated with basic living assistance and medical care services for disabled older adult individuals, LTCI alleviates the caregiving burden on both families and society. Furthermore, it enhances the capacity and specialization of older adult care services, fosters the development of integrated medical and nursing care, and facilitates the optimal allocation of older adult care resources. As a vital supplement to the social security system, LTCI is intricately linked to basic medical insurance, pension insurance, and other social security mechanisms, effectively bridging the financial gap in long-term care services for the disabled older adult and contributing to the establishment of a more comprehensive and multi-tiered social security framework (9, 10). As early as 2006, the Chinese government investigated the creation of a socialized service system, including LTCI for the older adult, to meet their long-term care needs and improve their quality of life. “Long-term care insurance” was first mentioned in government policy documents, but concrete plans were not submitted. During this period, Qingdao, Changchun, and Nantong spontaneously initiated LTCI practices. Qingdao pioneered the integration of medical and social resources by establishing a “long-term medical insurance system.” It is the first city in China to provide coverage for rural and demented older adult individuals (11). Nantong, on the other hand, created a “basic care insurance system” that separated long-term care services from medical resources and addressed the issue of “social hospitalization” (12). Changchun established a “smart long-term care” service through a platform that effectively links long-term care insurance with medical and work injury insurance. This led to information sharing and interconnectivity while avoiding the problem of duplicate payments (13).

By 2016, the Ministry of Human Resources and Social Security of China issued a proposal to launch a pilot LTCI program in 15 cities to accumulate experience and promote a nationwide universal care insurance system (14). As of October 2022, 145 million people were insured under China’s LTCI plan, with a total of 1.72 million people receiving long-term care insurance benefits. Details of the long-term care insurance policies in the pilot cities are presented in Table 1.

**Table 1:** Details of the long-term care insurance policies in pilot cities.

City	People covered		Beneficiaries		Funding sources		Reimbursement rates		
	Participants of urban employees’ health insurance and urban and rural health insurance	Only those who participate in the urban employees’ health insurance	Cover for severely disabled older adult only	Covering the older adult with moderate and severe disability	Employer and employee contributions only	Employer/employee contribution + financial assistance	More than 70% of the subsidy rate	70% of the subsidy rate	Less than 70% of the subsidy rate
Qiqihar		√	√		√				√
Changchun	√		√			√	√		
Chengde		√	√		√			√	
Qingdao	√			√		√	√		
Shanghai	√		√			√	√		
Nantong	√		√			√	√		
Suzhou	√		√			√			√
Ningbo		√	√		√				√
Guangzhou		√		√		√	√		
Anqing		√	√			√			√
Shangrao		√	√			√			√
Jingmen	√		√			√	√		
Chongqing		√	√		√				√
Chengdu		√	√			√			√
Shihezi	√		√			√	√		

City	Range of services			Service provision pathways			Scope of payment			Public-private partnerships	
	Provision of domiciliary care services	Primary care and rehabilitation services	Includes both of the above	Institutional care only	Provides institutional care and community care	Provides institutional care, community care and home care	Includes long-term care services only	Includes fees for nursing services, use of equipment and consumables	Full process costs	Operated by health insurance administrators only	Under-written by commercial insurers
Qiqihar	√				√		√			√	
Changchun		√		√					√	√	
Chengde	√				√		√			√	
Qingdao		√				√		√			√
Shanghai			√		√		√			√	
Nantong	√				√				√		√
Suzhou			√		√				√		√
Ningbo	√				√		√				√
Guangzhou			√		√				√		√
Anqing	√					√			√		√
Shangrao	√					√			√		√
Jingmen			√		√				√		√
Chongqing			√	√			√				√
Chengdu	√					√		√			√
Shihezi	√					√	√			√	

The pilot of LTCI in 15 cities across China has formed a basic institutional framework and issued a series of policy documents. A particular scale of participation was reached and local practical experiences and their characteristics were formed. As pilot trials have been underway for more than 5 years, the effectiveness of the policy needs to be fully verified and objectively judged.

As a social policy tool aimed at reducing the burden of family and social care and improving the quality of life of older people, the effectiveness of LTCI is affected by a variety of factors, including policy design, implementation process and resource allocation. In recent years, many countries and regions have begun to implement LTCI, and several scholars have conducted extensive empirical research on its effectiveness. Fong and Borowski (15) reviewed the development of Singapore’s LTCI policy through historical analysis and policy evaluation, noting that the CareShield Life programme sacrificed a certain amount of benefit in order to achieve broad coverage depth, but overall ensured the sustainability of LTCI. Zhang et al. (16) analysed the initiation process of China’s LTCI policy using a mixed data collection methodology and concluded that China’s LTCI policy was a successful case of policy transfer, which significantly improved the performance of China’s social security sector through the introduction of Germany’s LTCI experience. However, Feng et al. (17) point out that the current Chinese LTCI still faces a number of challenges, including the independence and sustainability of the financing pool, the balance between national priorities and local needs, coverage gaps and inequalities, the quality of services, and the lack of independent evaluation. In addition, LTCI as an important social policy tool, has been found to significantly improve the health status of older people. Zeng et al. (18), through an empirical study of an LTCI programme in Chengdu, China, found that the policy significantly reduced mortality and prolonged survival time among older people with disabilities, especially among specific subgroups (e.g., females, younger age, married, cared for by a family members and living in care-rich areas) the effect was more pronounced. In addition, a study by Wang and Feng (19) further confirmed that LTCI significantly reduced depressive symptoms and improved mental status scores and situational memory scores in older adults. These findings suggest that LTCI not only supports older adults financially, but also plays a positive role in psychological and physical health. Policy evaluation is key to improving policy effectiveness and achieving policy optimisation, and this paper intends to build on policy evaluation to explore whether the current LTCI pilot has had a positive impact on the health of middle-aged and older people in the pilot city.

This study makes two contributions to existing literature. First, since China’s LTCI system is relatively nascent, current research has largely focused on comparing policy options in pilot cities, investigating challenges related to policy implementation, and analyzing the impact of such policies on healthcare expenditure. Our study, on the other hand, seeks to augment the literature by examining the impact of LTCI on health outcomes. Second, our research employs micro panel data from the *China Health and Retirement Longitudinal Study* (CHARLS) to empirically evaluate the health effects of introducing LTCI to older adult individuals. Specifically, we utilized the pilot implementation of the LTCI system in 2016 as an exogenous policy shock to refine our understanding of policy implementation effects at the individual level.

## Reasons for building a long-term care insurance system in China

The China Development Research Foundation released a report on trends and policies for population aging in China. They predict that by 2035, China will have 310 million people aged 65 years and over, accounting for 22.3% of the total population, and nearly 380 million by 2050, accounting for 27.9% of the total population (20). As the proportion of the older adult population and life expectancy rises so does the risk of disability associated with advanced age, and the demand for long-term care increases across society. With a lower birth rate and traditional Chinese attitudes that old age and death are taboo, there is a shortage of people working in older adult services and care. The shortage of practitioners has resulted in the cost of long-term care rising beyond the reach of most older adult people, causing significant financial strain (21).

LTCI is an effective way of reducing the financial risks of long-term care. Insurance is a risk-sharing tool that reduces the risk of people paying catastrophic out-of-pocket costs through predictable and affordable premiums. While health insurance and pensions currently provide residents with protection against the risk of illness, the lack of a more effective system to protect the disabled older adult and long-term care arrangements through medical institutions will overtax healthcare resources. Furthermore, this is more likely to increase the average healthcare expenditure in China, making the burden on the currently unaffordable health insurance fund even heavier (22). The LTCI system, as an important social insurance arrangement after the “five insurance policies (pension, health, unemployment, work injury, and maternity insurance)” in China, provides room for economic instruments to deal with the risk of long-term incapacity that exists in society.

Two conclusions also exist among scholars on the health effects of LTCI. The first is based on the positive health effects of long-term care insurance. Kim and Mitra (3) found that older people in Korean LTCI beneficiary households tend to maintain better self-evaluations of their health than those in non-beneficiary households. A study by Ma et al. (23), guided by the concept of “value-based care,” found that LTCI policies improved the mental health of disabled clients and reduced their physical pain. The study conducted by Wang et al. (24) demonstrated a significant enhancement in overall health through an evaluation of self-rated health, activities of daily living (ADL), and instrumental activities of daily living (IADL). The second study focused on the negative health effects of long-term care insurance. It has further been suggested that health insurance and care delivery policies are not as important as expected (25, 26). Nemoto et al. (27), based on differences in physical vulnerability among people covered by LTCI, found that older people with poor health had poorer physical functioning after the implementation of LTCI.

Overall, studies exploring the impact of LTCI on health have yielded mixed results. This study examines the impact of a pilot LTCI policy on residents’ health in a pilot city in China, and dissects its effects using data from the waves of CHARLS.

## Materials and methods

The difference-in-difference model has been primarily used in the social sciences to assess the effects of policies. The rationale is to develop a counterfactual framework for assessing changes in the observed factor y under two scenarios: policy occurrence and non-occurrence. As a precondition, the y in the experimental and control groups was not significantly different before the policy shock. We suppose that an exogenous policy shock divides the sample into two groups: one for the experimental group subject to policy intervention and the other for the control group not subject to policy intervention. Thus, it is possible to consider the change in y in the control group before and after the policy as a situation in the experimental group that is not subject to the policy shock (a counterfactual result). By comparing the change in y in the experimental group (D1) with the change in y in the control group (D2), D1 minus D2 is the actual effect of the policy impact.

To test the impact of the introduction of the LTCI on the physical health of residents, it is common to judge the effect of the policy by comparing the differences in the physical health of residents before and after the introduction of LTCI. However, conclusions drawn from this simple regression may be inaccurate. Other factors may have influenced the health of residents before and after the introduction of LTCI; other health policies introduced during the same period may have improved the health of residents in non-pilot cities, thereby influencing the results. This potential impact was overlooked when the single-difference method was used. Therefore, using the Difference-in-Differences (DID) method for policy evaluation is both scientific and rigorous.

Of the 125 prefectures surveyed by CHARLS, 12 were piloting LTCI by 2015, creating a “quasi-natural experiment” for policy evaluation using the DID model. Specifically, individuals residing in cities that piloted LTCI in 2016 were designated as the experimental group, whereas those residing in cities that did not pilot LTCI in 2016 were designated as the control group. Application of the DID method presupposes that policy shocks are exogenous. However, the selection of pilot cities was not completely random and was based on various factors, such as geographical location, economic development, aging, and social security funds. Therefore, the choice of pilot cities may have affected the outcomes of this study and introduced endogeneity bias into the estimation results.

Considering this factor, this study employs propensity score matching (PSM) to construct a counterfactual control group that closely resembles the treatment group. This approach maximizes the reduction of sample selection bias and ensures a more rigorous and balanced dataset, thereby enhancing the accuracy of causal inference. Propensity score matching, as a data preprocessing method, compares the differential effects between experimental and control groups by matching and resampling, which allows the observable characteristics of the treatment and control groups to be as close as possible, thus overcoming the estimation bias due to sample self-selection (28). Based on PSM, DID was applied to further control factors that may affect the estimation results between the pilot and non-pilot cities and reduce the raw difference between the treatment and control groups. These variables include the gross per regional product, number of regional hospitals, hospital beds, and licensed doctors, and individual socioeconomic characteristics.

### Identification strategies

After controlling for other factors, the DID method allowed us to test whether there was a significant difference in self-rated health between respondents in the treatment group (pilot city) and the control group (non-pilot city) before and after the introduction of the LTCI pilot. The model can be expressed as Equation 1:


(1) $$Y_{it}^{PSM}=\beta_{0}+\beta_{1}DID_{it}+\beta_{2}control_{it}+\varphi_{i}+\gamma_{t}+\varepsilon_{it}$$

where $Y_{it}$ is the dependent variable, $DID_{it}$ is the core explanatory variable, $DID_{it}=treatment_{i}\times post_{t}$. In the sample period, if an individual’s city is listed as a pilot city, then = 1, otherwise 0; when t ≥ 2016, post =1, otherwise 0. $control_{it}$ denotes control variables that affect the dependent variable. $\varphi_{i}$ denotes individual fixed Effects. $\gamma_{t}$ denotes year fixed Effects. $\varepsilon_{it}$ denotes the error term. The estimated coefficients $\beta_{1}$ are the policy effects of interest in this paper, and if the policy is effective, it is significant.

### Selection of variables

#### Dependent variable

Improving the health status and quality of survival of care recipients is one of the roles of LTCI. Factors such as physical functioning, health status, and the quality of survival of older adult beneficiaries reflect the effectiveness of care services and the implementation of LTCI policies, which in turn affect LTCI service resources and the allocation strategy of financial resources. Scholars have also studied the impact of LTCI on the health of its beneficiaries. Huang et al. (29) showed that improving the health status of disabled older adult is an essential objective of the LTCI system and should be included as a core element in the evaluation of the effectiveness of LTCI home care services. Lee et al. (30) believed that LTCI helps delay the deterioration of beneficiaries’ activities of daily living through a timely assessment of their care status. Miguel et al. (31) concluded that LTCI reduces the level of depression and psychiatric behavioral symptoms in people under care, thus improving their quality of survival. Ju et al. (32) demonstrated through an experimental comparison that using home-visiting care services in long-term care insurance can reduce the risk of hospitalization. These studies suggest that LTCI has an impact on the health status of beneficiaries; therefore, respondents’ self-rated health was selected as the dependent variable.

#### Core explanatory variables

This study focuses on the interaction term DID of the LTCI pilot city; if the DID is significantly negative (a negative coefficient indicates improved health status since the questionnaire options are coded as 1 for very good health and 5 for bad health), then the policy is effective. The LTCI pilot was launched in 2016, this is a policy group dummy variable and a time dummy variable, and when the target is the pilot city = 1, otherwise = 0; when t ≥ 2016 = 1, and otherwise = 0.

#### Control variables

In addition to the LTCI pilot affecting respondents’ self-rated health, several other potential factors may also impact the dependent variables; therefore, the interference of these exogenous factors must be controlled when conducting the assessment. Drawing on relevant studies by Jing et al. (33) and Ma et al. (23), we selected the following control variables: (i) covariates at the individual level, including age, gender, household registration type, and marital status; (ii) socioeconomic status variables, such as educational attainment and availability of health insurance; and (iii) regional variables, such as gross per regional product, number of hospitals, number of hospital beds, and number of licensed doctors.

#### Data sources

This study uses four periods of microdata from the CHARLS database: 2011, 2013, 2015, and 2018. The CHARLS database is a comprehensive survey of individuals aged 45 years and older, encompassing the middle-aged and older adult populations. The survey project successfully interviewed approximately 20,000 respondents across 28 provinces in China by employing diverse sampling methods such as population proportional sampling. Its extensive data encompass microlevel information pertaining to families and individuals, including fundamental personal details, financial status, health conditions and functionality, access to medical care, and insurance coverage. This dataset serves as a valuable resource for facilitating high-quality data support. Much of the data for highly cited papers originates from this database, including those by Guo et al. (34), Zhao et al. (35), and Gong et al. (36). The reasons for selecting this database as the research sample are as follows: (i) Long-term care is mainly for older people who are at a greater risk of disability, and CHARLS provides research data specifically for middle-aged and older people aged 45 years and above. The survey respondents are closer to the subjects of this study. (ii) This paper studies the impact of the implementation of LTCI on the physical health of beneficiaries, and there are survey questions in CHARLS specifically for residents’ self-rated health and other related aspects, which can provide data for this study. (iii) In 2016, LTCI was implemented in several cities in China. The time points of the CHARLS data have a span that covers this study and provides a DID setting for this study. Its research sites are more numerous and can cover data from most pilot cities.

## Results

### Data description

The study combined data from four periods of CHARLS (2011, 2013, 2015, and 2018) and obtained a valid sample of 6,951 from pilot cities and 69,721 from non-pilot cities after assigning values to the data in segments. Descriptive information on the variables is presented in Table 2.

**Table 2:** Descriptive statistics for main variables.

Variables	Sample in the pilot cities		Sample in the non-pilot cities	
	Mean	SD	Mean	SD
Self-rated health	3.307	1.057	3.309	1.075
Educational attainment	3.281	1.830	3.464	1.974
Gender	1.521	0.500	1.523	0.499
Household registration type	0.983	0.701	0.984	0.678
Marital status	1.567	1.338	1.584	1.340
Health insurance	0.702	0.457	0.723	0.448
*Per Capita* GRP (regional)	3.045	1.082	2.460	1.144
Number of hospitals (regional)	3.321	0.976	2.836	1.158
Number of beds of hospitals (regional)	3.609	0.692	3.083	1.002
Numbers of licensed doctors (regional)	3.639	0.626	3.184	0.993
N (max)	6,999		62,738	
Number of cities covered by the sample	12		101	

### Propensity score matching (PSM)

#### Common support domain testing

**Figure 1:**
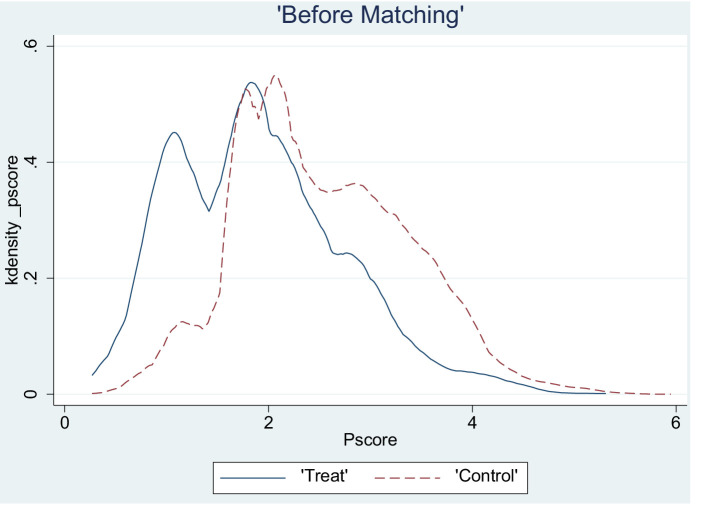
Density function plot after propensity score matching of individuals.

This study employed a logit model to estimate the propensity score of an LTCI pilot. A common support test was conducted to ascertain the validity and reasonableness of the PSM estimates. A probability density plot of the propensity score after matching is provided in Figure 1, which revealed that the propensity scores of the treatment and control groups were closely matched. Most of their values fell within a common range, indicating high-quality matching. The post-matching loss results (Table 3) demonstrate that the treatment and control groups still possessed 60,723 matched samples after the loss of 50 samples, implying good matching effectiveness. Additionally, the applicability test results (see Table 4) indicate a t-statistic of 2.74, which exceeds 2.58, implying significance at the 1% level.

**Table 3:** PSM matching results.

Variables	Treated	Controls	Difference	T-stat
Unmatched	3.302	3.304	−0.002	−0.17
Matched	3.302	3.243	0.059	2.74

**Table 4:** PSM applicability test.

Variables	Off support	On support	Total
Untreated	50	54,261	54,311
Treated	0	6,012	6,012
Total	50	60,273	60,323

#### Balance test

To ensure the reliability of the propensity score matching results, this study tested the balance of covariates. After matching, there were no significant systematic differences between individuals in the control and treatment groups concerning other covariates, except for differences in health insurance, number of hospital beds, and number of licensed doctors. The results of the balance test (see Table 5) show that the standardized bias of the explanatory variables was significantly reduced after sample matching and was less than the 10% required by the balance test. Based on the analysis of the results of the above tests, the use of propensity score matching is effective in reducing the differences in explanatory variables between the control and treatment groups and in eliminating estimation bias due to sample self-selection.

**Table 5:** The results of the balance test.

Variable	Unmatched	Mean		%reduct		*t*-test	
	Matched	Treated control		%bias	Bias	*t*	*p* > *t*
Educational attainment	U	3.2567	3.4422	−9.8		−7.00	0.000
	M	3.2567	3.2611	−0.2	97.6	−0.13	0.894
Gender	U	1.5259	1.527	−0.2		−0.16	0.872
	M	1.5259	1.5258	0	83	0.02	0.984
Marital status	U	1.5329	1.551	−1.4		−1.02	0.310
	M	1.5329	1.5012	2.4	−75.8	1.36	0.175
Health insurance	U	0.71507	0.73921	−5.4		−4.03	0.000
	M	0.71507	0.73782	−5.1	5.7	−2.80	0.005
Household registration type	U	0.9659	0.96995	−0.6		−0.44	0.660
	M	9,659	0.94165	3.5	−498.8	1.93	0.054
*Per Capita* GRP (regional)	U	3.0351	2.4399	53.3		38.29	0.000
	M	3.0351	3.0069	2.5	95.3	1.43	0.154
Number of hospitals (regional)	U	3.3105	2.8241	45.1		31.29	0.000
	M	3.3105	3.2864	2.2	95	1.33	0.185
Number of beds of hospitals (regional)	U	3.595	3.063	60.9		39.69	0.000
	M	3.595	3.5666	3.2	94.7	2.14	0.032
Numbers of licensed doctors (regional)	U	3.6354	3.1793	54.9		34.82	0.000
	M	3.6354	3.5998	4.3	92.2	2.98	0.003

##### Baseline regression results

This study estimated the direct effects of LTCI on residents’ health. As the pilot LTCI was rolled out in some cities across the country, a “quasi-natural experiment” was formed; therefore, the study used a DID approach to assess the net effect of LTCI on residents’ health. The regression results are presented in Table 6.

**Table 6:** Difference-in-difference model regression results.

Dependent variable	Self-rated health	Self-rated health
Independent variable	(1)	(2)
DID	−0.090*** (0.0261)	−0.093***(0.028)
Educational attainment		−0.032***(0.008)
Gender		−0.074 (0.083)
Marital status		0.016** (0.007)
Household registration type		0.026** (0.013)
Health insurance		0.002 (0.017)
*Per Capita* GRP (regional)		−0.009 (0.008)
Number of hospitals (regional)		0.015** (0.007)
Number of beds of hospitals (regional)		0.001 (0.011)
Numbers of licensed doctors (regional)		−0.011 (0.010)
Individual fixed effects	Yes	Yes
Time fixed effects	Yes	Yes
_cons	3.432***(0.007)	−3.609***(0.134)
N	66,833	60,323
CN	22,772	22,128
R^2^	0.0647	0.0667

*p < 0.1, **p < 0.05, ***p < 0.01.

In Table 6, Column 1 shows the estimated results when control variables are not included, and Column 2 shows the results when the control variables are included. After controlling for relevant variables, the implementation of LTCI in pilot cities is associated with a statistically significant increase of 0.093 levels in the self-rated health of surveyed residents. Notably, educational attainment, household registration, marital status, and the regional number of hospitals were all significant factors in this relationship.

The regression results showed that LTCI could effectively improve the self-rated health of the respondents. A possible reason for this is that LTCI, through the provision of care services by medical institutions, older adult care institutions, and families, has led to an improvement in the physical health of the older adult with disabilities, and some of those with less severe disabilities have begun to gradually regain their self-care abilities. Simultaneously, the reduction in financial pressure and family caregiving pressure also helps reduce the psychological burden on the disabled older adult and promote harmonious family relationships, which likewise contributes to physical recovery.

### Validity analysis of DID estimates

#### Parallel trend test

Performing the DID model requires satisfying the assumption of consistent trends in changes in the treatment and control groups before a policy event (37). The trend changes in the dependent variable should be consistent between pilot and non-pilot cities before the policy is implemented; if the health status of residents in the policy-implemented pilot cities is better (or worse) than that in the non-pilot cities, the presence of other factors cannot be ruled out, which will inevitably lead to inaccurate model estimates. Therefore, a parallel trend test was conducted. The more common method of testing for parallel trends is direct graphical observation; however, because of the small number of data periods in the study, graphical observation is less obvious. Therefore, the regression method was adopted in this study. The empirical equation can be written as Equation 2:


(2) $$Y_{it}=\alpha_{0}+\alpha_{k}treated\sum_{j=2013}^{2018}year_{jt}+Z_{it}+\varepsilon_{it}$$

where $year_{jt}$ is an annual dummy variable, and observations are set to 1 for the year after policy implementation and 0 for other years. All other variables were consistent with those in the baseline model.

We examined data from two periods before and one period after the introduction of the LTCI pilot program in 2016. As shown in Table 7, none of the regression results for 2013 and 2015 (before the onset of the policy) were significant, indicating that before the implementation of the LTCI pilot, the trends in the treatment and control groups were consistent and not significantly different. In contrast, after 2016, the self-rated health of respondents in the treatment group improved significantly compared to that of the control group; thus, the sample data passed the parallel trend test required for estimation using the DID method.

**Table 7:** Parallel trend test.

Dependent variable	Self-rated health	
	Coef.	*P* > | *t* |
DID_2013	−0.056	0.126
DID_2015	−0.031	0.394
DID_2018	−0.123	0.001***
Time fixed effects	Yes	
Individual fixed effects	Yes	
Time fixed effects	Yes	
N	60,323	
CN	22,128	
R2	0.0668	

*p < 0.1, **p < 0.05, ***p < 0.01.

#### Counter-trend test

In addition to the parallel trend test, this study conducts a counter-trend test by assuming the timing of policy implementation. In addition to the LTCI implementation affecting residents’ health status, other policies or unobservable factors may have caused changes in the dependent variable. This change may not be linked to LTCI, thus affecting the reliability of the previous conclusions. To exclude such factors, this study sets the policy event of the implementation of the LTCI pilot in the sample cities before 2015 as the counter-trend test. Based on this hypothesis, 2011 and 2013 were pre-policy implementations, and 2015 and 2018 were post-policy implementations. As stated in the previous analysis, the DID method presupposes no significant change in respondents’ self-rated health prior to the policy event; thus, if the policy event is set before 2015, the estimated coefficients of the core variables are theoretically insignificant. If the results do not meet the expected judgement, there are indeed some underlying unobservable factors that influence respondents’ self-rated health; the dependent variable is not influenced only by the facilitative effect of LTCI implementation. The corresponding estimation results are presented in Table 8. Based on Table 8, the estimated coefficients of the core variables are not significant and are in line with expectations. Therefore, other potentially unobservable factors influencing respondents’ self-rated health were excluded.

**Table 8:** Counter-trend test.

Dependent variable	Self-rated health
Independent variable	(1)
DID	−0.034(0.028)
Control variables	Yes
Individual fixed effects	Yes
Time fixed effects	Yes
N	60,323
CN	22,128
R2	0.0665

*p < 0.1, **p < 0.05, ***p < 0.01.

#### Heterogeneity analysis

The previous analyses suggest that long-term care insurance has a significant health-promoting effect on residents. We would like to know further whether there is a policy effect of long-term care insurance across regions, genders, and income groups, and if so, does this effect differ? Our study further analyses the heterogeneity of the effect of long-term care insurance.

#### Regional heterogeneity analysis

The sample was divided into eastern, central, and western cities by combining the characteristics of economic development level and geographical location. A list of cities is attached as an Appendix A. We usually think of eastern China as the most economically developed, central China as the second most developed, and western China as the weakest. Table 9 presents the empirical results of the impact of the LTCI policy on the health status of residents in cities in different regions. Overall, the policy implementation has a boosting effect on the health of residents in western cities, which is significant at the 1% level, and on the health of residents in central cities at the 5% level; however, there is no significant boosting effect in eastern cities.

**Table 9:** Heterogeneous results of regions.

Variables	Eastern cities	Central cities	Western cities
DID	−0.059	−0.093**	−0.158***
Individual fixed effects	Yes	Yes	Yes
Time fixed effects	Yes	Yes	Yes
Control variables	Yes	Yes	Yes

*p < 0.1, **p < 0.05, ***p < 0.01.

The observed disparities may stem from the demographic and socioeconomic characteristics of eastern cities, which, as major hubs for population inflow, tend to have younger resident populations and a relatively smaller proportion of aging individuals. Consequently, the demand for LTCI in these regions is comparatively less urgent than in cities located in central and western regions. Furthermore, a larger population that brings more diverse needs, making the current relatively single pilot scheme slightly less effective.

Table 10 presents data on the levels of economic development and the distribution of healthcare resources across selected regions. As illustrated, eastern regions benefit from more favorable conditions, evidenced by indicators such as regional Gross Domestic Product, per capita disposable income of urban residents, the number of public hospitals, healthcare technicians, healthcare technicians per 1,000 people, hospital beds, and healthcare consultations and treatments.

**Table 10:** Economic level and distribution of medical resources by region.

Variables	Regional Gross National product (trillion)	Per capita disposable income of urban residents (yuan)	Quantity of public hospitals	Number of health technicians	Number of health technicians per 1,000 population	Number of beds in medical and health institutions	Number of patients treated in medical and health institutions (person/time)
Eastern cities	62.2	58459.9	4,418	5,089,295	8.38	3,719,811	4,133,148,304
Central cities	26.65	42733.4	3,520	3,333,357	7.95	3,172,304	2,216,864,577
Western cities	25.7	42173.3	3,808	3,235,226	8.45	2,857,818	2,066,263,430

Additionally, as economically advanced areas, eastern cities have long offered a wider range of aging support options, including home care, combined medical care services, and nursing homes. This established infrastructure may partially explain why the introduction of LTCI has not resulted in significant health improvements among residents in these regions.

In contrast, cities in central and western regions, which historically provide fewer care services for individuals with disabilities, have demonstrated more pronounced health benefits following the implementation of LTCI pilot programs. The policy appears to address critical service gaps in these regions, thereby yielding more substantial improvements in residents’ health outcomes.

#### Gender heterogeneity analysis

The sample is first divided into two subgroups, male and female, according to gender, aiming to reveal the potential heterogeneous effects of LTCI on the health status of urban residents of different genders. As shown in Table 11, the results of the empirical analyses clearly present the differential effects of the LTCI policy in improving the health status of men and women.

**Table 11:** Heterogeneous results of genders.

Variables	Male	Females
DID	−0.083***	−0.048
Individual fixed effects	Yes	Yes
Time fixed effects	Yes	Yes
Control variables	Yes	Yes

*p < 0.1, **p < 0.05, ***p < 0.01.

The heterogeneity analysis reveals a significant positive impact of LTCI on self-rated health among male respondents, as indicated by a DID coefficient of −0.083 (*p* = 0.012). This suggests that the implementation of LTCI has contributed to improved health perceptions among older adult men. Several factors may explain this finding. Compared to women, men tend to exhibit weaker health management behaviors and lower healthcare-seeking tendencies in later life. The introduction of LTCI likely facilitated access to formal care services, thereby enhancing their overall well-being.

In contrast, while the DID coefficient for women is also negative (−0.048), indicating a similar trend, the effect does not reach statistical significance (*p* = 0.127). This discrepancy may stem from inherent gender differences in self-care ability, lifestyle habits, and baseline health conditions. Women generally exhibit stronger self-sufficiency in daily activities, healthier lifestyles, and a greater inclination toward preventive healthcare measures. Consequently, they may have been less reliant on LTCI services, resulting in a more limited observable impact on their SRH. Moreover, given that women tend to have longer life expectancies and a higher prevalence of chronic conditions, the benefits of LTCI may take longer to manifest, making short-term improvements less pronounced.

#### Low-income group heterogeneity analysis

Further, this study divided the interviewed population into low-income and non-low-income groups based on China’s official poverty criterion (annual income of less than RMB 3,000), with a view to accurately assessing the mechanism of LTCI’s effect on the health status of individuals with different economic status. Table 12 details the results of the empirical analyses for these two income levels.

**Table 12:** Heterogeneous results of income groups.

Variables	Economically disadvantaged Individuals	Non-disadvantaged individuals
DID	−0.0141	−0.0138
Individual fixed effects	Yes	Yes
Time fixed effects	Yes	Yes
Control variables	Yes	Yes

*p < 0.1, **p < 0.05, ***p < 0.01.

The heterogeneity analysis comparing economically disadvantaged individuals (annual income ≤ 3,000 RMB) and non-disadvantaged individuals reveals that the implementation of LTCI has not resulted in statistically significant improvements in self-rated health for either group. Specifically, the DID coefficient for the economically disadvantaged group is −0.0141 (*p* = 0.832), while for the non-disadvantaged group, it is −0.0138 (*p* = 0.692). Although both coefficients are negative, suggesting a potential positive effect of LTCI on self-rated health, the lack of statistical significance implies that the observed changes may not be robust. Several explanations could account for these findings.

For the economically disadvantaged group, the absence of a significant effect may be attributed to practical barriers such as limited awareness of the policy, difficulties in accessing services, and an inadequate supply of formal care providers, despite the financial support offered by LTCI. These barriers may have hindered effective utilization, thereby attenuating its impact. Moreover, health outcomes in this group are influenced by a complex interplay of factors beyond financial assistance, including social support networks, health literacy, and access to broader healthcare services, which may not be directly addressed by LTCI alone.

Similarly, the non-disadvantaged group does not exhibit a significant improvement in self-rated health following LTCI implementation, likely due to their relatively better pre-existing health status and greater access to healthcare resources. Since individuals in this group are more capable of managing their health independently, they may have had lower demand for LTCI services, leading to minimal observable effects.

## Discussion

Our research adopted a quasi-natural experimental approach, leveraging longitudinal microdata from the CHARLS database spanning several years to evaluate the impact of the LTCI pilot program on residents’ health status. By applying the DID method, we isolated the causal effects of LTCI implementation, accounting for potential confounding factors and regional variations. The results of our analysis revealed a significant positive effect of the LTCI policy on residents’ health status. However, the mechanisms underlying this association require further investigation.

Based on existing theoretical frameworks and literature, it has been confirmed that long-term care has the potential to mitigate the irrational use of health insurance funds by reducing delayed discharges and bed blockers. Additionally, it has been shown to improve the health of those receiving care, which, in turn, reduces health service utilization and alleviates pressure on fund operations (38, 39). We suggest that LTCI policies affect residents’ health status through three channels. First, LTCI provides living care, medical care, rehabilitation training, and psychological counselling services for the disabled, which help them restore their health while rebuilding their self-care capacity, ultimately improving their quality of life. Second, some cities have experimented with home care programs as part of their LTCI coverage. Home care helps alleviate the isolation of older adult people living in residential care facilities. This ensures communication with family members and neighbors while receiving adequate care and promotes the recovery of physical health with the help of good psychological energy. Finally, institutional care can provide more professional care for the disabled older adult than the old forms of family and nursing care. Quality care services not only meet the basic needs of the disabled older adult but also include advanced services such as medical care and rehabilitation training, which help them recover as much as possible.

Our findings align with the results of previous studies conducted by Liu et al. (37) and Kim and Mitra (3). Lei et al. (40) demonstrated improvements in self-reported health and a reduced mortality risk among older adults with initial care needs in China, whereas Kim and Mitra (3) found similar health improvements among households that benefited from LTCI in Korea. In contrast, the study by Lei et al. (40) places greater emphasis on exploring the relationship between LTCI and factors such as care needs, household financial burdens, and healthcare expenditures among older adults. Similarly, the research conducted by Kim and Mitra (3) investigates the health improvements observed in households benefiting from LTCI, particularly those with lower savings, in comparison to households that do not benefit from such coverage.

Our study further highlights regional disparities in the impact of LTCI implementation on residents’ health status, with central and western cities showing a stronger effect than eastern pilot cities. The phenomenon suggests that variations in economic development levels and the distribution of healthcare resources across regions significantly influence the effectiveness of LTCI in enhancing residents’ health. This underscores the need for tailored approaches and targeted interventions to address specific needs and challenges faced by different regions. The gender-based heterogeneity emphasizes the necessity for nuanced and differentiated strategies in the design of long-term care policies to ensure that LTCI evolves into a more precise, equitable, and inclusive system. Given the substantial benefits observed among men, future policy measures should incorporate more proactive health promotion strategies targeting older adult males, encouraging early engagement in preventive healthcare and self-management practices. Conversely, for women, expanding the scope of LTCI to include more targeted interventions—such as enhanced chronic disease management and psychological support services—could maximize its efficacy. More broadly, the lack of statistically significant effects for economically disadvantaged and non-disadvantaged groups suggests that while LTCI may contribute to improved self-rated health, its full potential may only become evident over an extended period. Future research should focus on evaluating the long-term health implications of LTCI and exploring complementary policy measures to maximize its effectiveness across diverse demographic and socioeconomic groups.

Policy pilots in China play a distinctive role in advancing the country’s economic and social development, fostering policy and institutional innovation, and are of immense significance in establishing a robust long-term care framework. However, there are concerns and challenges associated with this approach. For instance, all pilot programs rely on surpluses from existing social health insurance schemes (41, 42). This approach entails risks due to the rapid growth of healthcare spending in China, which exceeds real GDP growth by four percentage points annually at a rate of 12% (43). Moreover, the UEBMI and URRBMI funds have experienced losses in many areas and need to access reserves (44, 45). Furthermore, China’s current social medical insurance programs, particularly the URRBMI in low-income regions, offer limited coverage, which results in high out-of-pocket medical expenses. Diverting Medicare funds to support LTCI may exacerbate this issue.

To ensure a sustainable and equitable financing framework for LTCI, it is imperative to explore diversified funding models that incorporate contributions from individuals, enterprises, social organizations, and government entities. These models may include mechanisms such as commercial LTCI, corporate and social group donations, and government financial transfers. It is essential for central financial authorities to adopt a coordinated approach to interregional payment transfers, particularly targeting areas that bear a disproportionate burden of long-term care costs, thereby ensuring an equitable distribution of resources. Additionally, multifaceted funding channels should be developed, drawing on public welfare and charitable funds, social donations, and revenues from welfare lotteries. Charitable organizations can play a pivotal role in alleviating the financial burden of LTC on individuals by offering financial assistance or implementing targeted funding programs.

To further enhance the robustness of the funding base, it is necessary to implement a dynamic adjustment mechanism for funding bases and contribution rates. Such a mechanism should aim to reasonably determine the aggregate funding requirements, ensuring a balance between income and expenditure, with a modest surplus to safeguard financial stability.

It is important to highlight the considerable variation in reimbursement limits for LTC expenses across different regions and populations in China. This variability, while rooted in the disparate levels of economic development and service provision capacity among pilot regions for LTCI, poses challenges to achieving equitable outcomes. Such disparities, being structural in nature, are unlikely to be resolved in the short term. However, the findings of Kim and Mitra (3) underscore that LTCI has a particularly pronounced positive impact on health outcomes among lower-income groups, further emphasizing the necessity of addressing equity within the LTCI system.

To ensure fairness and inclusivity in LTCI services, the development of the LTCI service system must be tailored to local conditions, adopting regionally differentiated strategies. In the more economically advanced eastern regions, there should be a greater emphasis on leveraging market mechanisms to enhance service delivery. Conversely, in the central and western regions, efforts should focus on strengthening the supportive roles of public older adult care institutions, grassroots health organizations, and the foundational contribution of family care. Such a localized and stratified approach will not only promote equity in LTCI services but also contribute to the establishment of a comprehensive and accessible basic older adult care system for disabled older adult individuals.

Furthermore, the adoption of diversified payment models for long-term care LTCI holds significant potential for increasing public participation, broadening insurance coverage, and ensuring stable and sustainable financing. Currently, more than half of China’s pilot cities utilize a service-based payment model, approximately one-third employ a hybrid model combining monetary payments with service payments, while the remaining cities rely solely on monetary payment models (46). Unlike the individualistic cultures prevalent in Europe and the United States, Confucianism emphasizes the concept of “raising children for old age” and prioritizes home-based care over institutional care. This cultural ideology is particularly evident in Japan and South Korea, both of which belong to the Confucian cultural sphere. In these societies, filial piety and family responsibilities are deeply ingrained, resulting in a caregiving model where family members typically serve as the primary caregivers, and home-based care remains the predominant form of older adult support. Consequently, long-term care insurance policies in these countries often integrate a combination of cash subsidies, care service vouchers, and tax incentives for children who assume caregiving responsibilities, thereby reinforcing family-based care structures (47). In contrast, Singapore provides long-term care benefits primarily in the form of cash, offering recipients flexibility in its use. These benefits are not restricted to older adult care services but can also be allocated to medical expenses and other essential needs, allowing for greater autonomy in financial decision-making (48).

To build a robust and inclusive LTCI system in China, it is crucial to prioritize the development of flexible and diversified payment mechanisms. Incorporating family care into the LTCI payment structure can further enhance the system’s adaptability, enabling tailored treatment options and more responsive payment solutions to meet diverse care requirements effectively. Such flexibility would not only improve individual satisfaction but also strengthen the system’s capacity to deliver equitable and comprehensive long-term care services.

## Conclusion

The findings of this study provide valuable insights into the evolving landscape of LTCI policies in China and their impact on the health status of residents. Actively advancing the implementation of LTCI represents a critical and strategic initiative in public policy, particularly within the context of an aging society, as it addresses pressing demographic challenges and contributes to the promotion of equitable and sustainable healthcare systems.

Although this study contributes to the assessment of the policy effects of LTCI, it also has some weaknesses. Although this study contributes to evaluating the policy effects of LTCI, it also has certain limitations. Due to the availability of data and the short duration of the LTCI pilot, this study only captured data from the first period after its implementation. We present only a detailed analysis of the dependent variable of population health and do not provide a comprehensive understanding of the contribution and impact of other aspects of LTCI. A new batch of pilot cities is committing to the LTCI pilot program, and with an increasing amount of research data being added, we can expect future empirical research on the effects of LTCI policies to be more rigorous and comprehensive.

In conclusion, although the findings of this study provide evidence of the positive impact of LTCI policies on residents’ health status, further research and ongoing monitoring are necessary to refine and optimize these initiatives. By addressing regional disparities, enhancing program sustainability, and incorporating comprehensive policy frameworks, policymakers can ensure the continued improvement of LTCI programs, ultimately contributing to the overall well-being of the population.

## Data Availability

Publicly available datasets were analyzed in this study. This data can be found at: http://charls.pku.edu.cn/.
