# Supplementary material for: Effects of long-term care insurance on health: a study of the middle-aged and older adult in pilot cities in China
Source: Front Public Health. 2025 May 19;13:1498105. doi: 10.3389/fpubh.2025.1498105 (PMC12127334; doi:10.3389/fpubh.2025.1498105)
Supplement: Supplementary file 1 [file Supplementary_file_1.docx]

**Appendix A**. A list of cities classified as eastern, central, and western regions

| Eastern Cities | Central Cities | Western Cities |
| --- | --- | --- |
| Beijing | **Shanxi Province** | **Inner Mongolia Autonomous Region** |
| Tianjin | Taiyuan | Hohhot |
| Chongqing | Datong | Baotou |
| **Hebei Province** | Yangquan | Wuhai |
| Shijiazhuang | Changzhi | Chifeng |
| Tangshan | Jincheng | Tongliao |
| Qinhuangdao | Shuozhou | Erdos |
| Handan | Jinzhong | Hulunbuir |
| Xingtai | Yuncheng | Bayannur |
| Baoding | Xinzhou | Ulanqab |
| Zhangjiakou | Linfen | Xilin Gol |
| Chengde | Lvliang | Hinggan |
| Cangzhou | **Jilin Province** | **Guangxi Zhuang Autonomous Region** |
| Langfang | Changchun | Nanning |
| Hengshui | Jilin | Liuzhou |
| **Liaoning Province** | Siping | Guilin |
| Shenyang | Liaoyuan | Wuzhou |
| Dalian | Tonghua | Beihai |
| Anshan | Baishan | Fangchenggang |
| Fushun | Songyuan | Qinzhou |
| Benxi | Baicheng | Guigang |
| Dandong | **Heilongjiang Province** | Yulin |
| Jinzhou | Harbin | Bose |
| Yingkou | Qiqihar | Hezhou |
| Fuxin | Jixi | Hechi |
| Liaoyang | Hegang | Laibin |
| Panjin | Shuangyashan | Chongzuo |
| Tieling | Daqing | **Sichuan Province** |
| Chaoyang | Yizhong | Chengdu |
| Huludao | Jiamusi | Zigong |
| Shanghai | Qitaihe | Panzhihua |
| **Jiangsu Province** | Mudanjiang | Luzhou |
| Nanjing | Heihe | Deyang |
| Wuxi | Suihua | Mianyang |
| Xuzhou | **Anhui Province** | Guangyuan |
| Changzhou | Hefei | Suining |
| Suzhou | Wuhu | Neijiang |
| Nantong | Bengbu | Leshan |
| Lianyungang | Huainan | Nanchong |
| Huai 'an | Ma 'anshan | Meishan |
| Yancheng | Huaibei | Yibin |
| Yangzhou | Tongling | Guang 'an |
| Zhenjiang | Anqing | Dazhou |
| Taizhou | Huangshan | Ya 'an |
| Suqian | Chuzhou | Bazhong |
| **Zhejiang Province** | Fuyang | Ziyang |
| Hangzhou | Suzhou | Liangshan |
| Ningbo | Lu 'an | Garze |
| Wenzhou | Bozhou | **Guizhou Province** |
| Jiaxing | Chizhou | Guiyang |
| Huzhou | Xuancheng | Liupanshui |
| Shaoxing | **Jiangxi Province** | Zunyi |
| Jinhua | Nanchang | Anshun |
| Quzhou | Jingdezhen | Bijie |
| Zhoushan | Pingxiang | Tongren |
| Taizhou | Jiujiang | Qiandongnan |
| Lishui | Xinyu | Qiannan |
| **Fujian Province** | Yingtan | **Yunnan Province** |
| Fuzhou | Ganzhou | Kunming |
| Xiamen | Ji 'an | Qujing |
| Putian | Yichun | Yuxi |
| Sanming | Fuzhou | Baoshan |
| Quanzhou | Shangrao | Zhaotong |
| Zhangzhou | **Henan Province** | Lijiang |
| Nanping | Zhengzhou | Pu 'er |
| Longyan | Kaifeng | Lincang |
| Ningde | Luoyang | Chuxiong |
| **Shandong Province** | Pingdingshan | **Tibet Autonomous Region** |
| Jinan | Anyang | Lhasa |
| Qingdao | Hebi | Shigatse |
| Zibo | Xinxiang | Qamdo |
| Zaozhuang | Jiaozuo | Nyingchi |
| Dongying | Puyang | Sannan |
| Yantai | Xuchang | Nagqu |
| Weifang | Luohe | **Shaanxi Province** |
| Jining | Sanmenxia | Xi 'an |
| Tai 'an | Nanyang | Tongchuan |
| Weihai | Shangqiu | Baoji |
| Rizhao | Xinyang | Xianyang |
| Laiwu | Zhoukou | Weinan |
| Linyi | Zhumadian | Yan 'an |
| Dezhou | **Hubei Province** | Hanzhong |
| Liaocheng | Wuhan | Yulin |
| Binzhou | Huangshi | Ankang |
| Heze | Shiyan | Shangluo |
| **Guangdong Province** | Yichang | **Gansu Province** |
| Guangzhou | Xiangyang | Lanzhou |
| Shaoguan | Ezhou | Jiayuguan |
| Shenzhen | Jingmen | Jinchang |
| Zhuhai | Xiaogan | Baiyin |
| Shantou | Jingzhou | Tianshui |
| Foshan | Huanggang | Wuwei |
| Jiangmen | Xianning | Zhangye |
| Zhanjiang | Suizhou | Pingliang |
| Maoming | Enshi | Jiuquan |
| Zhaoqing | **Hunan Province** | Qingyang |
| Huizhou | Changsha | Dingxi |
| Meizhou | Zhuzhou | Longnan |
| Shanwei | Xiangtan | **Qinghai Province** |
| Heyuan | Hengyang | Xining |
| Yangjiang | Shaoyang | Haidong |
| Qingyuan | Yueyang | Haidong |
| Dongguan | Changde | **Ningxia Hui Autonomous Region** |
| Zhongshan | Zhangjiajie | Yinchuan |
| Chaozhou | Yiyang | Shizuishan |
| Jieyang | Chenzhou | Wuzhong |
| Yunfu | Yongzhou | Guyuan |
| **Hainan Province** | Huaihua | Zhongwei |
| Haikou | Loudi | **Xinjiang Uygur Autonomous Region** |
| Sanya |  | Urumqi |
| Sansha |  | Karamay |
| Danzhou |  | Turpan |
|  |  | Hami |
|  |  | Aksu |
